# The Coefficient of Variation of Step Time Can Overestimate Gait Abnormality: Test-Retest Reliability of Gait-Related Parameters Obtained with a Tri-Axial Accelerometer in Healthy Subjects

**DOI:** 10.3390/s20030577

**Published:** 2020-01-21

**Authors:** Shunrou Fujiwara, Shinpei Sato, Atsushi Sugawara, Yasumasa Nishikawa, Takahiro Koji, Yukihide Nishimura, Kuniaki Ogasawara

**Affiliations:** 1 Department of Neurosurgery, Iwate Medical University, 1-1-1 Idaidori, Yahaba, Iwate 028-3695, Japan; ssato.neuro@gmail.com (S.S.); asuga@iwate-med.ac.jp (A.S.); yasnishi777@yahoo.co.jp (Y.N.); thkoji@hotmail.com (T.K.); kuogasa@iwate-med.ac.jp (K.O.); 2 Institute for Open and Transdisciplinary Research Initiative, Osaka University, 3-1 Yamadaoka, Suita, Osaka 565-0871, Japan; 3 Department of Rehabilitation Medicine, Iwate Medical University, 1-1-1 Idaidori, Yahaba, Iwate 028-3695, Japan; ynishi@iwate-med.ac.jp

**Keywords:** gait assessment, tri-axial accelerometer, CV, healthy subjects, test-retest

## Abstract

The aim of this study was to investigate whether variation in gait-related parameters among healthy participants could help detect gait abnormalities. In total, 36 participants (21 men, 15 women; mean age, 35.7 ± 9.9 years) performed a 10-m walk six times while wearing a tri-axial accelerometer fixed at the L3 level. A second walk was performed ≥1 month after the first (mean interval, 49.6 ± 7.6 days). From each 10-m data set, the following nine gait-related parameters were automatically calculated: assessment time, number of steps, stride time, cadence, ground force reaction, step time, coefficient of variation (CV) of step time, velocity, and step length. Six repeated measurement values were averaged for each gait parameter. In addition, for each gait parameter, the difference between the first and second assessments was statistically examined, and the intraclass correlation coefficient (ICC) was calculated with the level of significance set at *p* < 0.05. Only the CV of step time showed a significant difference between the first and second assessments (*p* = 0.0188). The CV of step time also showed the lowest ICC, at <0.50 (0.425), among all parameters. Test–retest results of gait assessment using a tri-axial accelerometer showed sufficient reproducibility in terms of the clinical evaluation of all parameters except the CV of step time.

## 1. Introduction

Walking is naturally performed by humans without any deficits, and it is one of the indexes that show normality of motor and/or cognitive function in healthy subjects and abnormality in patients [1,2,3,4,5,6]. In patients with neurodegenerative diseases such as Parkinson’s or Huntington’s disease, freezing of gait or reduction of gait performance is often observed [7,8,9]. For assessing the gait in post-stroke patients during the follow-up period, a special portable stride analyzer was used in a previous work [2]. This device consisted of special insoles with compression sensors in shoes, and the insole was connected to a mobile data collection box worn on the belt. The other study to assess the gait in patients with subcortical capsular encephalopathy also used a similar device [10]. On the other hand, an electronic walkway that detects the spatial and temporal characteristics of footfalls during gait was used in the assessment of walking behavior in multiple sclerosis [11,12]. Then, a camera-based device was also used for gait assessments in patients with spinal cord injury in addition to multiple sclerosis [1,13]. Various techniques for quantitative gait assessment have been proposed in previous works; however, gait performance is difficult to assess, requiring the use of special or large machines and facilities in clinical scene and/or multicenter trials [1,10,11,12,13,14,15]. In practice, qualitative assessments have often been performed without any devices, but with the use of clinical scores [16,17], and no standard device for quantitative gait assessment has been established.

The portable devices for gait assessment, particularly a tri-axial accelerometer [18,19,20,21], that have been developed recently exhibit an accuracy equal to that of large devices such as treadmills in the assessment of gait performance and are used in practical and clinical studies [18,19,20,21,22,23,24]. Tri-axial accelerometers have the advantage of easy attachment to the body or foot of subjects with a belt and the estimation of various gait parameters such as movements of the body trunk, step time, and ground reaction force from the acceleration wave dataset in the three axial directions during walking. On the other hand, the reproducibility of the accelerometer in healthy subjects remains unclear. In the present study, we investigated whether gait-related parameters obtained by a tri-axial accelerometer are reliable in terms of reproducibility by performing test-retest gait measurement in healthy subjects.

## 2. Materials and Methods

### 2.1. Subjects

**Figure 1:**
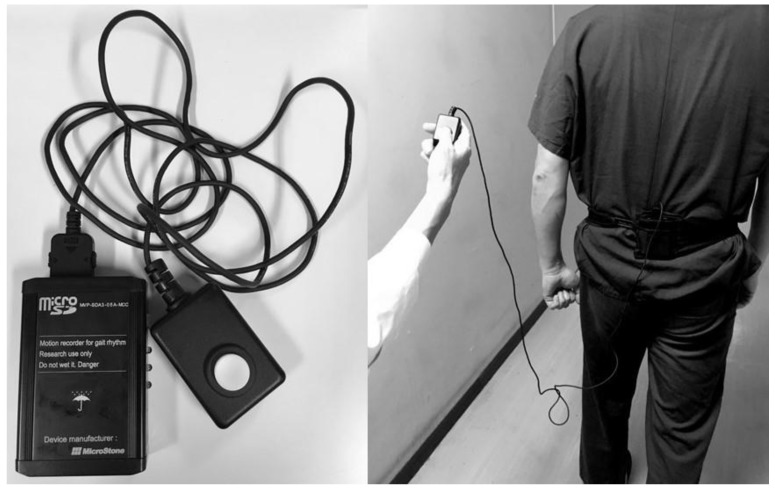
Tri-axial accelerometer (MG-M1110) with a switch cable for marking points of a 10-m walking interval for the dataset (**left**) and the accelerometer fixed at the L3 level by a nylon belt in a subject (**right**).

All subjects participated in this study between July 2017 and October 2017 after providing written informed consent and primary medical check interviewing history of disorders, age and height by authors (S.S. and Y.N.). The inclusion criteria were as follows: >20 but <60 years of age; no history of brain-related disorders, including surgical operation, irradiation, stroke, infection, remarkable atrophy, or demyelinating disease; no history of hypertension, diabetes mellitus, atrial fibrillation, pulmonary disease, leukoaraiosis, no musculoskeletal deficits or the other diseases showing gait abnormalities without neurological deficits [24,25,26,27]. In the first stage, each subject performed a 10-m walk six times with a tri-axial accelerometer (MG-M1110-HW, LSI Medience, Tokyo, Japan) fixed at the L3 level of the subject by a nylon belt (Figure 1).

The device can measure tri-axial (vertical, anteroposterior, and mediolateral) acceleration by detecting limb and trunk movements at a sampling rate of 100 Hz during step-in and kick-off motions. The tests were performed on a 30-m straight walkway in our hospital. All subjects were instructed by an author (S.F.) for walking at each usual pace and they walked 16 m, including 3 m before the starting point and 3 m after the end point, as intervals to obtain the 10-m walking dataset in Table 1. To mark the 10-m segment of the dataset, an operator (S.F.) pushed a button connected to the accelerometer with a cable at both the start and end points while following the subject. The second test was also performed with the same subjects under the same conditions (the tri-axial accelerometer, an operator, and the walkway) within a 3-month period at least 1 month after the first evaluation. The statistical comparison of two datasets, each showing 10% standard deviation relative to each mean value and 10% mean difference to an averaged value of the two mean values, requires the examination of more than 28 subjects with alpha and beta levels less than 0.01 [28], indicating Type I error and Type II error, respectively. Thus, we determined that the present study required more than 28 subjects in order to compare the first and second tests.

### 2.2. Data Analysis

**Figure 2:**
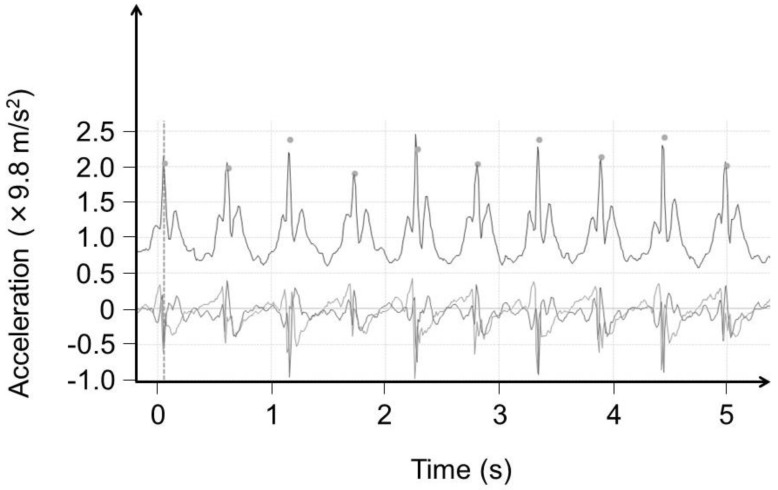
Wave dataset obtained using a tri-axial accelerometer during walking.

From each tri-axial acceleration wave dataset of six repetitions of the 10-m walk measurement, the following nine gait parameters were calculated using commercial software (LSI Medience): assessment time (s); number of steps (step); stride time (s; time from initial contact of one foot to subsequent contact of the same foot); cadence (step/m); ground floor reaction (×9.8 m/s^2^); step time (s; time from initial contact of one foot to initial contact of the other foot); coefficient of variation (standard deviation/mean × 100) of step time (%; CV); velocity (m/min); and step length (cm). To calculate these gait parameters, pre-processing, called “step extraction”, which marks each wave indicating a step in the tri-axial acceleration wave dataset (Figure 2), was needed.

By a pre-processing “step extraction”, markers were automatically placed on top of each wave, indicating one step.

During pre-processing with the software, the number of steps can be automatically estimated from each 10-m walking wave dataset. If the number of steps was clearly low (e.g., less than half of the number calculated using the other 10-m walking wave datasets in the same subject) by missing the wave peak due to the limitation of the peak detection algorithm, the subject was excluded from the analysis in this study without the manual error correction for the same conditions and protocols for all wave dataset. Finally, the six values from each gait parameter obtained from the six measurements were averaged, and the averaged value was defined as the representative value of the parameter in a subject. The protocol of this study was reviewed and approved by the institutional ethics committee.

### 2.3. Statistical Analysis

The differences in each gait parameter between the first and second assessments were examined using the Wilcoxon signed-rank test. To validate the reproducibility in two gait assessments with a tri-axial accelerometer, the intraclass correlation coefficient (ICC) was also calculated in each gait parameter. Grading of the ICC was defined as follows: excellent, ICC ≥ 0.9; good, 0.7 ≤ ICC < 0.9; moderate, 0.5 ≤ ICC < 0.7; and poor, ICC < 0.50. Subsequently, a Bland-Altman plot was performed to confirm the tendency of the relationship between the two measurements in each parameter. Furthermore, the correlation between height and each gait parameter at the 1st test was statistically examined with Spearman’s correlation coefficient for confirming the effect from the individual factor. All statistical analyses were performed on MedCalc ver. 17.9.7 (MedCalc Software bvba, Ostend, Belgium) with a significance level of *p* < 0.05.

## 3. Results

**Figure 3:**
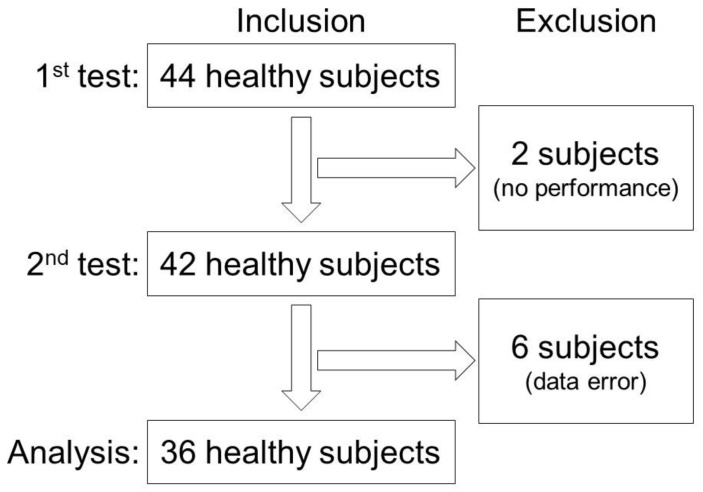
Flowchart for including the subjects.

Forty-four subjects were included in this study. Two of the 44 subjects could not perform the second test within 3 months. The other 42 subjects performed the 10-m walk at both stages; however, some step waves of the six measurements in six subjects could not be appropriately extracted for each 10-m walk wave, indicating that step extraction errors during pre-processing for gait analysis occurred because of the deterioration of the waveform and the limitation of the wave extraction algorithm. Finally, 36 subjects (Figure 3) (21 men and 15 women; mean age, 35.7 ± 9.9 years; range, 22–58 years) were able to complete both the first and second analyses (mean interval, 49.6 ± 7.6 days; range, 40–65 days).

**Figure 4:**
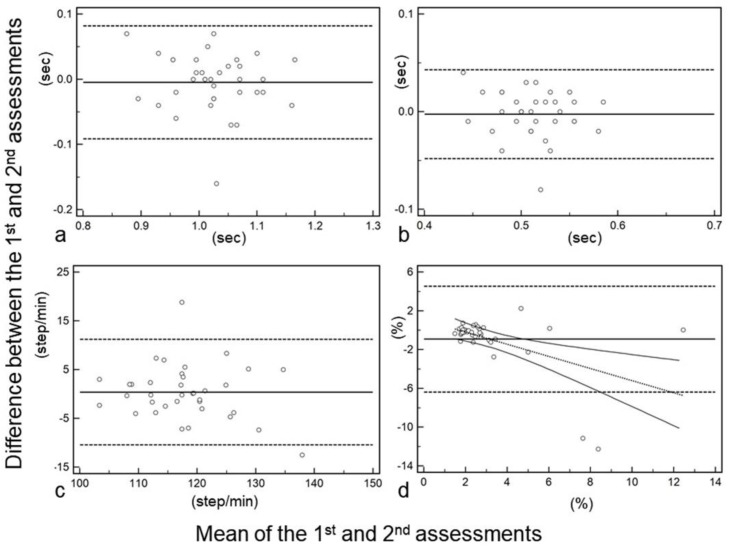
Bland-Altman plots of the gait parameters, showing good intraclass correlation coefficient (ICC; (**a**) stride time, (**b**) step time, (**c**) cadence) and poor ICC ((**d**) coefficient of variation).

Only the CV showed a significant difference between the first and second measurements (median CV: first, 2.16; second, 2.50; *p* = 0.0188), while the other parameters showed no significant differences (Table 1). Among all nine gait parameters, stride time (ICC: 0.803), step time (0.788), and cadence (0.784) showed good correlation with a high ICC of ≥0.70. The number of steps (0.685), step length (0.663), ground reaction force (0.615), velocity (0.598), and assessment time (0.565) showed a moderate ICC between 0.50 and 0.70. The ICC of the CV indicated poor correlation and was the lowest value, being <0.50 (0.425) in all the parameters (Table 1). The Bland-Altman plot in the CV showed a negative trend, with the mean of the first and second assessments being larger, while those in the other parameters showed no trends. The plot of the parameters shows good ICCs in Figure 4a–c, and the CV shows poor ICC in Figure 4d.

Height significantly correlated with number of steps (ρ, *p*-value and the 95% confidential interval: −0.358, 0.0323, −0.614 to −0.0328), stride time (0.468, 0.0040, 0.164 to 0.690), cadence (−0.467, 0.0041, −0.690 to −0.163), step time (0.483, 0.0028, 0.184 to 0.701) or step length (0.356, 0.0331, 0.0311 to 0.613). On the other hand, no significant correlation was observed between height and assessment time (ρ, *p* value and the 95% confidential interval: 0.0312, 0.8567, −0.300 to 0.356), ground force reaction (−0.139, 0.4174, −0.447 to 0.198), CV (0.0464, 0.7883, −0.287 to 0.369), or velocity (−0.0340, 0.8441, −0.359 to 0.298).

## 4. Discussion

Portable devices for quantitative gait analysis have been developed, and these devices have the advantage in clinical scenarios of needing only a walkway, rather than any large space or facility [18,19,21,22,23,29,30,31]. In the present study, we validated the reproducibility of a tri-axial accelerometer used in gait assessment by performing test-retest measurements within a 3-month period 1 month after the first evaluation. All gait parameters except the CV showed adequate reproducibility for practical clinical use, with no significant differences and with practical ICCs between test-retest measurements. The present findings suggest that the tri-axial accelerometer can sufficiently evaluate gait by using just the device and an operator, and without large machines and experts to analyze the dataset.

Three gait parameters (stride time, step time, and cadence) showed good ICC in the present study. In a previous work, the parameters showed no significant differences between controls (patients showing transient ischemic stroke/asymptomatic carotid artery stenosis) and patients without symptoms 1 month after stroke but significantly changed in patients with symptoms after stroke as compared with controls [2]. These three parameters may be more robust than the other gait parameters because they only change in cases that show severe deficits. Furthermore, the device we used may have more sensitivity in such parameters than previous devices; thus, it may also indicate a remarkable robustness in these three parameters.

By contrast, the parameters that show moderate ICC (number of steps, step length, ground reaction force, velocity, and assessment time) may change depending on the condition and/or intention of the subjects participating in the gait measurement. We presume that velocity and assessment time can change more easily than the other parameters because the same pace is difficult for subjects to retain between the first and second measurements, even if an operator carefully performs the measurements with healthy subjects under the same conditions. Thus, when we use these parameters for clinical research, it may be insufficient for identifying the gait abnormalities in the pathological groups to use the standard cutoff values at the 95% confidential intervals (*p* = 0.05) from the healthy groups because the sensitivity to detect the abnormalities may be low with the cutoff values, especially for assessing improvement of ambulation by velocity [32] or assessment time [12,17], as described in a previous study. We have to pay attention to the use of such gait parameters.

A previous report indicated that the CV was significantly larger in patients with Parkinson’s or Huntington’s disease (HD) than in controls, and that the CV was the best predictor of HD [9]. On the other hand, the averaged value of each gait parameter during gait assessment showed no significant difference between the pathological and control groups in the previous study. The CV showed a significant difference in the present study and a poor ICC compared to that of the healthy subject group. This result indicates that the CV in the previous work may potentially underestimate the gait performance in the pathological group. With the high accuracy of the accelerometer used in the present study, we could identify the significant variation in the CV observed even in the healthy subjects. Therefore, the averaged value of each gait parameter during gait assessment may show the significance in the pathological group if the tri-axial accelerometer that shows high sensitivity is used.

The present study has some limitations. First, the sample size is not so large. Second, the gait dataset may include errors due to a slight slipping of the nylon belt from the L3 level of the subject. Third, the 10-m distance was marked by manually pushing a button connected to the accelerometer with a cable in the present study. Only one operator performed the measurements for gait assessment; therefore, interrater reliability remains unclear. In future studies, a comparison of the tri-axial accelerometer as used in the present study with an accelerometer based on an infrared system (as proposed in a previous study [31]) tell us the difference in the accuracy between manual and automatic procedures.

## 5. Conclusions

In the test-retest gait assessment using a tri-axial accelerometer, a reproducibility sufficient for clinical research was observed in all parameters except the CV. The present results suggest careful evaluation of the CV because it may potentially overestimate gait disturbance in the pathological group owing to the comparably low reproducibility. Portable accelerometers can assess gait performance noninvasively and with practical accuracy without the need for other huge machines. In future works, the devices may be used for long-term gait assessment over a few days in the elderly and in patients with neurodegenerative and/or spinal disease because subjects only need to attach the device to their back with a belt.

## Figures and Tables

**Table 1:** Median and intra-correlation coefficient for each parameter at and between first and second 10-m walks in healthy subjects (*n* = 36).

	1st	95% CI	2nd	95% CI	*p* Value *	ICC	95% CI
Stride time [sec]	1.02	1.01–1.05	1.03	0.99–1.05	0.689	0.803	0.647–0.894
Cadence [step/min]	119	115–120	117	114–121	0.765	0.784	0.616–0.884
Step time [sec]	0.505	0.500–0.523	0.515	0.500–0.523	0.697	0.788	0.624–0.886
Number of steps [step]	13.8	13.5–14.2	13.9	13.5–14.2	0.765	0.685	0.462–0.827
Step length [cm]	72.3	69.7–73.8	72.0	70.7–73.6	0.981	0.663	0.429–0.813
Ground reaction force [×9.8 m/s^2^]	0.360	0.330–0.383	0.355	0.327–0.373	0.980	0.615	0.361–0.784
Velocity [m/min]	85.3	82.1–87.1	84.3	82.3–86.3	0.753	0.598	0.339–0.773
Assessment time [s]	7.04	6.89–7.33	7.13	6.96–7.30	0.831	0.565	0.293–0.752
Coefficient of variance [%]	2.16	1.98–2.57	2.50	2.15–2.95	0.0188	0.425	0.129–0.655

ICC: intra-correlation coefficient; CI: confidential interval. * examined using Wilcoxon signed-rank test.
